# Sox17 protects human brain microvascular endothelial cells from AngII-induced injury by regulating autophagy and apoptosis

**DOI:** 10.1007/s11010-023-04838-5

**Published:** 2023-09-02

**Authors:** Yanyan Wang, Marong Fang, Qiannan Ren, Wei Qi, Xinli Bai, Nashwa Amin, Xiangjian Zhang, Zhenzhong Li, Lihong Zhang

**Affiliations:** 1 Department of Neurology, The Second Hospital of Hebei Medical University, No 215 Heping West Road, Shijiazhuang, 050000 Hebei Province China; 2 The Key Laboratory of Neurology (Hebei Medical University), Ministry of Education, Shijiazhuang, China; 3 Institute of System Medicine, Zhejiang University School of Medicine, Zhejiang University, Hangzhou, China; 4 Department of Gastroenterology, The Second Hospital of Hebei Medical University, Shijiazhuang, China; 5 Department of Pediatrics, The Second Hospital of Hebei Medical University, Shijiazhuang, China; 6 Department of Zoology, Faculty of Science, Aswan University, Qism Aswan, Egypt

**Keywords:** Intracranial aneurysm, Sox17 gene, Human brain microvascular endothelial cells, Angiotensin II, Autophagy, Apoptosis

## Abstract

Intracranial aneurysm (IA), is a localized dilation of the intracranial arteries, the rupture of which is catastrophic. Hypertension is major IA risk factor that mediates endothelial cell damage. Sox17 is highly expressed in intracranial vascular endothelial cells, and GWAS studies indicate that its genetic alteration is one of the major genetic risk factors for IA. Vascular endothelial cell injury plays a vital role in the pathogenesis of IA. The genetic ablation of Sox17 plus hypertension induced by AngII can lead to an increased incidence of intracranial aneurysms had tested in the previous animal experiments. In order to study the underlying molecular mechanisms, we established stable Sox17-overexpressing and knockdown cell lines in human brain microvascular endothelial cells (HBMECs) first. Then flow cytometry, western blotting, and immunofluorescence were employed. We found that the knockdown of Sox17 could worsen the apoptosis and autophagy of HBMECs caused by AngII, while overexpression of Sox17 had the opposite effect. Transmission electron microscopy displayed increased autophagosomes after the knockdown of Sox17 in HBMECs. The RNA-sequencing analysis shown that dysregulation of the Sox17 gene was closely associated with the autophagy-related pathways. Our study suggests that Sox17 could protect HBMECs from AngII-induced injury by regulating autophagy and apoptosis.

## Introduction

Intracranial aneurysm (IA), a localized dilation of the intracranial arteries, is a common cerebrovascular disease. IA rupture can lead to life-threatening subarachnoid hemorrhage (SAH) [1]. At present, the underlying molecular mechanisms of IA have not been fully elucidated. Previous studies have shown that vascular endothelial cells are essential for maintaining the structure and integrity of blood vessels [2, 3], and intracranial vascular endothelial cell injury plays a vital role in the progression of IA [4, 5]. Hypertension, one of the risk factors of intracranial aneurysms [6], mainly leads to vascular endothelial cell damage [7]. In the in vivo and in vitro experiments, angiotensin II (AngII) is widely used as a canonical inducer in hypertension models to trigger apoptosis and autophagy in vascular endothelial cells [8–10] and is an enhancer of vascular aging [9]. AngII can also be used as an inducer to establish an in vitro model of intracranial aneurysms [11]. According to a series of genome-wide association studies (GWAS) based on IA patient populations, the Sox17 gene was identified as one of the susceptible genes of IA [5, 12, 13]. The Sox (SRY Related-HMG box) gene family harbors an HMG box DNA-binding domain associated with SRY-related transcription factors. The 20 members of the Sox family are evolutionarily conserved and can be divided into several subclasses according to the DNA-binding domain homology, which plays an essential role in regulating tissue differentiation and organoid morphologies [14]. Therefore, mutations, deletions, or abnormal expression of the Sox gene can lead to dysplasia or other serious diseases. The SoxF subclass, consisting of Sox7, Sox17, and Sox18, promotes the cell fate determination of endothelial cells [15, 16]. Sox17 is highly expressed in the intracranial vascular endothelial cells [4], and is essential for neoangiogenesis during development and arterial integrity in adulthood [17, 18]. Previous studies have shown that knockdown of Sox17 can lead to impaired cell proliferation of human aortic endothelial cells, and genetic ablation of Sox17 combined with AngII treatment can cause vascular morphological abnormalities and endothelial dysfunction in mice, resulting in an increased occurrence and rupture of intracranial aneurysms in mice [4]. However, the underlying molecular mechanism of how Sox17 alteration affects vascular endothelial cell injury [19], particularly under hypertension conditions, has not been elucidated.

Vascular endothelial growth factor (VEGF) is a secretory protein that acts specifically on vascular endothelial cells and is one of the most potent stimulators of angiogenesis [20]. Sox17, a critical component of the SoxF subclass, can potentiate angiogenesis by targeting the VEGF signaling pathway [21]. Decreased expression of VEGF can lead to autophagy of vascular endothelial cells, eventually leading to endothelial cell apoptosis [22]. Nevertheless, whether the alterations of Sox17 affect cell apoptosis and autophagy of vascular endothelial cells is unclear. Apoptosis is a process of programmed cell death, and numerous studies have shown a synergistic relationship between apoptosis and autophagy [23]. Autophagy is the lysosomal degradation of cellular components under stress conditions to provide the energy needed for cell survival [24, 25]. Studies have shown that apoptosis and autophagy are essential for maintaining cellular homeostasis in mammalian cells [26, 27]. Disrupt balance between apoptosis and autophagy may result in vascular aging and related diseases [28].

In the current study, we used AngII-stimulated human brain microvascular endothelial cells (HBMECs) as a cell model of hypertension to explore the effect of Sox17 on apoptosis and autophagy of HBMECs, which might reveal the underlying molecular mechanisms of how Sox17 affects IA formation.

## Materials and methods

### Cell culture

The human brain microvascular endothelial (HBMEC) cell line was purchased from Otwo Biotech (Cat# HTX1843, China, RRID: CVCL_U985). The cells were cultured in DMEM solution (Gibco, USA) supplemented with 10% fetal bovine serum (Gibco, USA) and 1% penicillin–streptomycin (Gibco, USA) under normoxic conditions (37 ℃, 5% CO_2_) in the incubator. The culture medium was changed every two days. When the cell density reached more than 80%, the cells were washed twice in 0.01 M phosphate buffer saline (PBS) (HyClone) and passaged by using 0.25% trypsin–EDTA (Gibco, USA).

### AngII-induced hypertension HBMEC model

To establish a hypertension model, HBMECs were treated with AngII as previously reported [8]. AngII (Calbiochem, USA) was dissolved in ddH_2_O and diluted with culture medium to obtain the working concentrations. The HBMECs were seeded in 96-well plates at a density of 5 × 10^4^/mL and cultured for 24 h. Afterward, the cells were treated with different concentrations of AngII (10^–9^, 10^–8^, 10^–7^, 10^–6^, 10^–5^ mol/L) at 37 °C in 5% CO_2_ for another 24 h. Finally, the appropriate concentration was selected based on the cell viability assay results.

### Cell viability assay

Cell viability rate was measured using the Cell Counting Kit-8 (CCK-8, Dojindo, Japan). After treatment with AngII, the cells were incubated with culture medium containing 10% CCK-8 solution at 37 °C for 2.5 h. The absorbance at 450 nm was then measured. Cell viability of the treatment groups was expressed as the percentage of viable cells normalized to that of the control group. Experiments were repeated three times. According to the result of cell viability, the most suitable concentration of AngII was chosen for subsequent experiments.

### Sox17 overexpression and knockdown

The cells were seeded in 6-well plates at a density of 5 × 10^4^/mL. Sox17 knockdown shRNA (Sox17-Kd), Sox17 overexpression vector (Sox17-Ad), and their corresponding control vectors (vector-Kd and vector-Ad) were transfected into cells using lentivirus transfection reagent (Shanghai Genechem China). The cells in the 6-well plate were incubated with a transfection mixture (1 mL per well) for 12 h, after which the medium was replaced by normal culture medium. Green fluorescence protein (GFP) was expressed in the successfully transfected cells. So the infection efficiency was determined by counting GFP-expressed cells under a fluorescence microscope 72–96 h after virus infection. More than 90% of the cells expressing GFP would be considered a successful infection. After the virus infection, the cells in each group were selected with puromycin (Solarbio 1 μg/mL) for 48 h in a CO_2_ incubator.

### Western blot analysis

For protein extraction, the AngII-treated cells were collected with ice-cold RIPA buffer containing protease and phosphatase inhibitors (Roche, Switzerland). The protein concentrations were measured using a BCA protein quantitative detection kit (Auragene Bioscience, China). Samples were adjusted to the same concentration (2 μg/μL) and denatured with 5 × SDS loading buffer in boiling water for 10 min. Total protein was separated by electrophoresis (200 V) until the loading dye reached the bottom of the gel and transferred to polyvinylidene fluoride (PVDF) membrane (Immobilon) at 300 mA for 60 min. Afterward, the PVDF membranes were blocked with TBST-containing 5% skim milk for 3 h at room temperature. The primary antibodies were used overnight at 4 °C and as the following: anti-GAPDH antibody (1:1000, CST), anti-Sox17 antibody (1:1000, CST), anti-LC3B antibody (1:1000, Novus), anti-P62 antibody (1:1000, CST), anti-Beclin1 antibody (1:1000, CST),anti-Bax antibody (1:500, Boster), anti-Bcl-2 antibody (1:1000, Abclonal). On the next day, after washing three times with TBST buffer, the membranes were incubated with HRP-conjugated secondary antibody at room temperature for 2 h. The protein bands incubated with enhanced chemiluminescence (ECL) detection kit were exposed in the ChemiDoc Touch Imaging System and quantified by Image Lab software. All experiments were performed three times.

### Immunofluorescence staining

The cultured cells were seeded onto coverslips and fixed in 4% paraformaldehyde at room temperature (RT) for 40 min. After washing three times in PBS, cells were permeabilized with 0.2% TritonX-100 in 0.01 M PBS for 15 min. Subsequently, the fixed cells were exposed to the blocking solution (5% BSA) and were incubated with primary antibody (rabbit anti-LC3B 1:250 dilution, Novus) in PBST overnight at 4℃. After three washes in PBS, the cells were incubated with anti-rabbit Alexa Fluor 594 secondary antibody (1:500,EARTHOX, USA) in 0.01 M PBST at room temperature for 1.5 h. Afterward, a mounting medium containing DAPI (VECTASHIELD, USA) was dripped onto the slides and covered with coverslips. The slides were observed under a laser scanning confocal microscopy (Olympus FV1200, Japan). Images were captured at 200 × magnification. Five sections of the captured images were selected from each slide for analysis and quantified with Image J software. Relative density = IOD/area. As a negative control, PBS was used to replace the primary antibody.

### Detection of apoptosis by flow cytometry analysis

The cells were incubated for 48 h in a CO2 incubator. Cells were digested by 0.25% trypsin, and cell density was adjusted to 1 × 10^6^ /mL. Apoptosis of HBMEC cells was determined by flow cytometry (CytoFLEX LX; Beckman Coulter, Brea, CA, USA) using PE Annexin-V Apoptosis Detection Kit I (BD Pharmingen, USA).The cells were resuspended in 100μL 1 × annexin-binding buffer and then stained with 5μL of PE Annexin-V and 7-AAD for 15 min at room temperature in the dark. After the incubation, 400μL 1 × annexin-binding buffer was added, and stained cells were measured by flow cytometry. The rate of apoptotic cells was analyzed by using CytExpert software.

### Transmission electron microscopy

The treated cells were digested, centrifuged with serum-containing PBS washing buffer, fixed overnight with 2.5% glutaraldehyde 4 °C, then fixed with 1% osmic acid, stained with 2% uranyl acetate, then dehydrated with ethanol increased by gradient, acetone infiltration and finally embedded in epoxy resin, and cut into electron microscopy sample, visualized with transmission electron microscope machine viewing.

### Transcriptome sequencing

Total RNA of Sox17 overexpression and knockdown cell lines and their corresponding control groups was extracted using Trizol reagent (Beyotime, China), and a genome-wide transcriptomics analysis was conducted by LC-Bio Technology Co., Ltd (Hangzhou, China). The RNA amount and purity of each sample was quantified using NanoDrop ND-1000. The RNA integrity was assessed by Bioanalyzer 2100 and confirmed by electrophoresis with denaturing agarose gel. Then use the Magnesium RNA Fragmentation Module and SuperScript™ II Reverse Transcriptase for reverse transcription to obtain cDNA, which were next used to synthesise U-labeled second-stranded DNAs with E. coli DNA polymerase I, RNase H and dUTP Solution. The average insert size for the final cDNA library was 300 ± 50 bp after PCR. At last, we performed the 2 × 150 bp paired-end sequencing (PE150) on an illumina Novaseq™ 6000. Fastp software were used to control the quality of offline original data, We used HISAT2 to map reads to the reference genome of Homo sapiens GRCh38. The mapped reads of each sample were assembled using StringTie with default parameters. The differentially expressed mRNAs were selected with fold change > 2 or fold change < 0.5 and *p*-value < 0.05 by R package edge R or DESeq2. Hierarchical clustering heatmap was generated by pheatmap R package. Then the KEGG enrichment analysis was performed according to the differentially expressed mRNAs. Significantly enriched KEGG pathways were analyzed using the DAVID software and selected based on a threshold *p*-value ≤ 0.05. Volcano plot was constructed using the ggVolcano Rpackage.

### Statistical analysis

Data are expressed as mean ± SEM (*n* = 3). Differences between two groups were calculated by an unpaired *t*-test in SPSS 23.0 software. One-way ANOVA and two-way ANOVA followed by Tukey's post hoc test in SPSS 23.0 software were used to determine the statistical significance between multiple groups when appropriate. Histograms were generated using GraphPad Prism8. *p* < 0.05 was considered statistically significant.

## Results

### Treatment of AngII impaired HBMEC cell viability

**Fig. 1:**
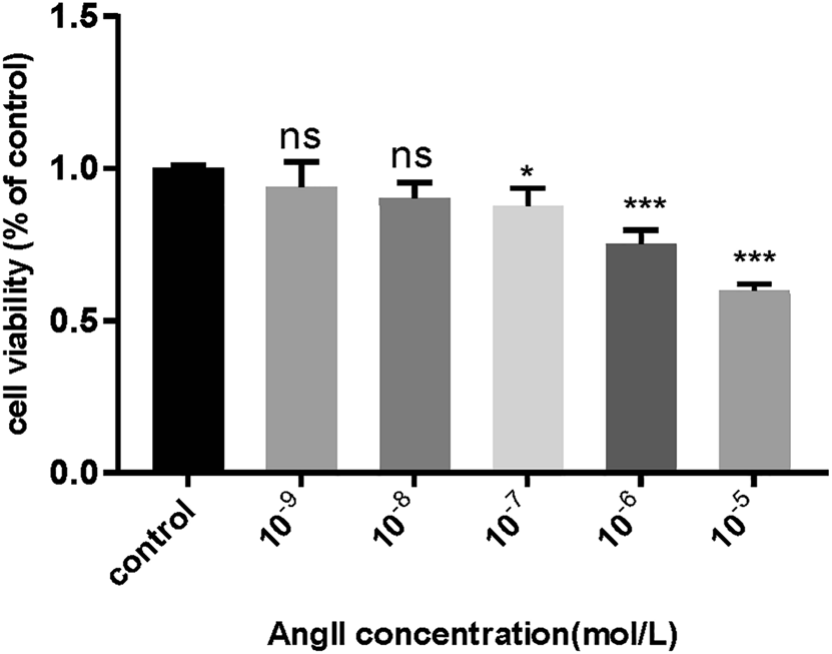
The effect of different concentrations of AngII on cell viability. Cell viability was measured by CCK-8 assay. Values are expressed as mean ± SEM (*n* = 3).***p < 0.001, *p < 0.05. One-way ANOVA with Tukey's multiple comparisons test was used. ns, not significant

As the concentration of AngII increases, the cell viability gradually decreases (Fig. 1). Compare with control group, we observed an approximately 50% decline in cell viability after 10^–5^ mol/L AngII treatment. This concentration was chosen as the intervention concentration for further experiments.

### Construction of Sox17 stable cell line in HBMEC cell

**Fig. 2:**
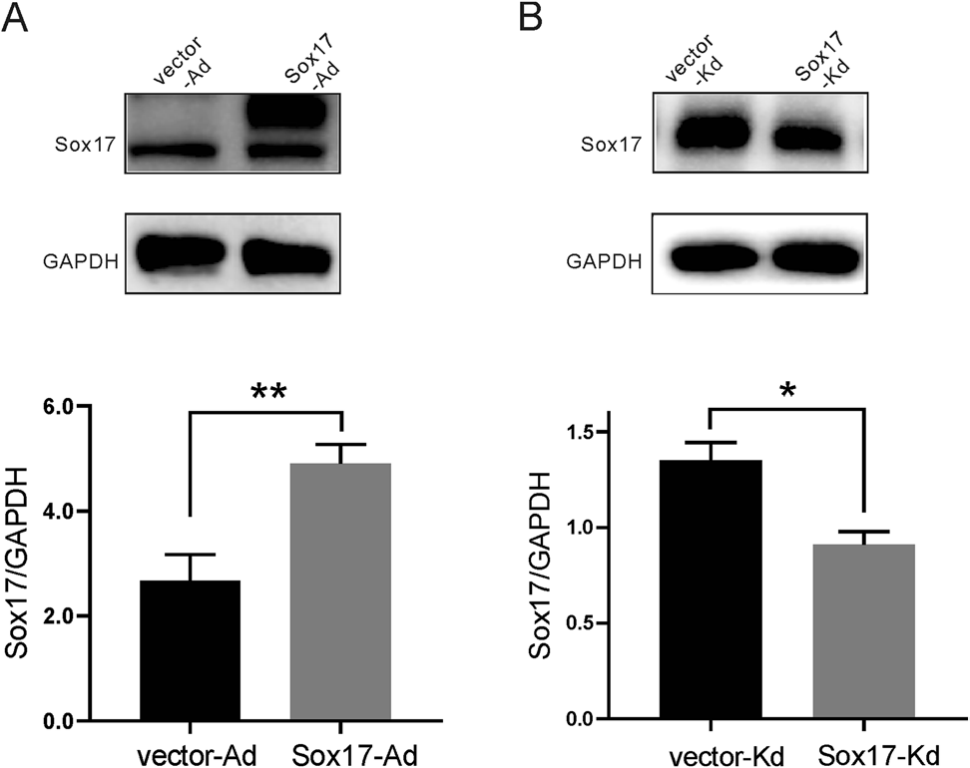
The establishment of Sox17 stable cell lines. **A** The human brain microvascular endothelial (HBMEC) cells were infected by the Sox17 overexpression (Sox17-Ad) and the corresponding EGFP control (vector-Ad) lentivirus. Western blotting was used to examine the overexpression of Sox17. **B** HBMEC cells were infected by the lentivirus encoding different Sox17 shRNA(Sox17-Kd) and their control vector (vector-Kd). Western blotting was used to examine the knockdown of Sox17. Data were expressed as mean ± SEM (*n* = 3). **p < 0.01, * *p* < 0.05, *t*-test

Based on western blot analysis, Sox17 expression is up-regulated significantly after overexpression of Sox17 gene (Fig. 2A), and Sox17 expression was decreased by about 33% after the knockdown of Sox17 gene (Fig. 2B).

### Effect of Sox17 on regulating the apoptosis of HBMEC

**Fig. 3:**
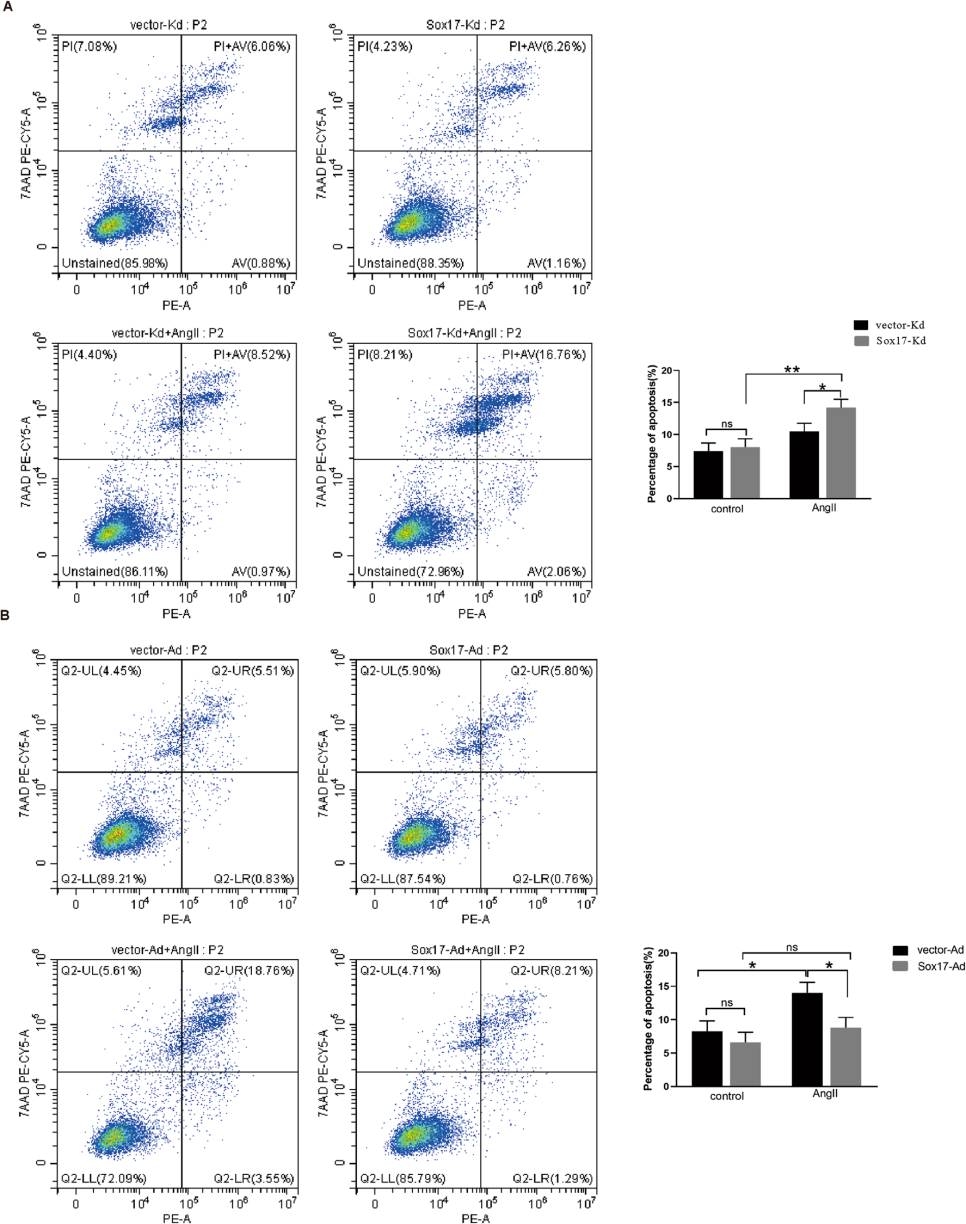
Flow cytometry was used to monitor the effect of Sox17 on modulating cell apoptosis. **A** HBMEC with different treatments were stained with fluorescein isothiocyanate Phycoerythrin (PE)-conjugated Annexin-V and 7-Amino-Actinomycin (7-AAD). Flow cytometry was used to analyze the percentage of apoptosis after knockdown of Sox17 in the absence or presence of AngII. **B** Flow cytometry was used to analyze the percentage of apoptosis after overexpression of Sox17 in the absence or presence of AngII. Data were expressed as mean ± SEM (*n* = 3). *p < 0.05, ** *p* < 0.01, two-way ANOVA with Tukey's multiple comparisons test. *ns* not significant, *AngII* angiotensin II, *control* without angiotensin II

According to the flow cytometry assay, neither overexpression nor knockdown of Sox17 significantly affects on the apoptotic rate of HBMEC (Fig. 3A&B). Under the stimulation by AngII, the apoptotic rate of the Sox17-Kd group significantly increased compared with that of the control group (Fig. 3A), while the Sox17-Ad group showed the opposite result (Fig. 3B).

### Effect of Sox17 on apoptosis-related proteins

**Fig. 4:**
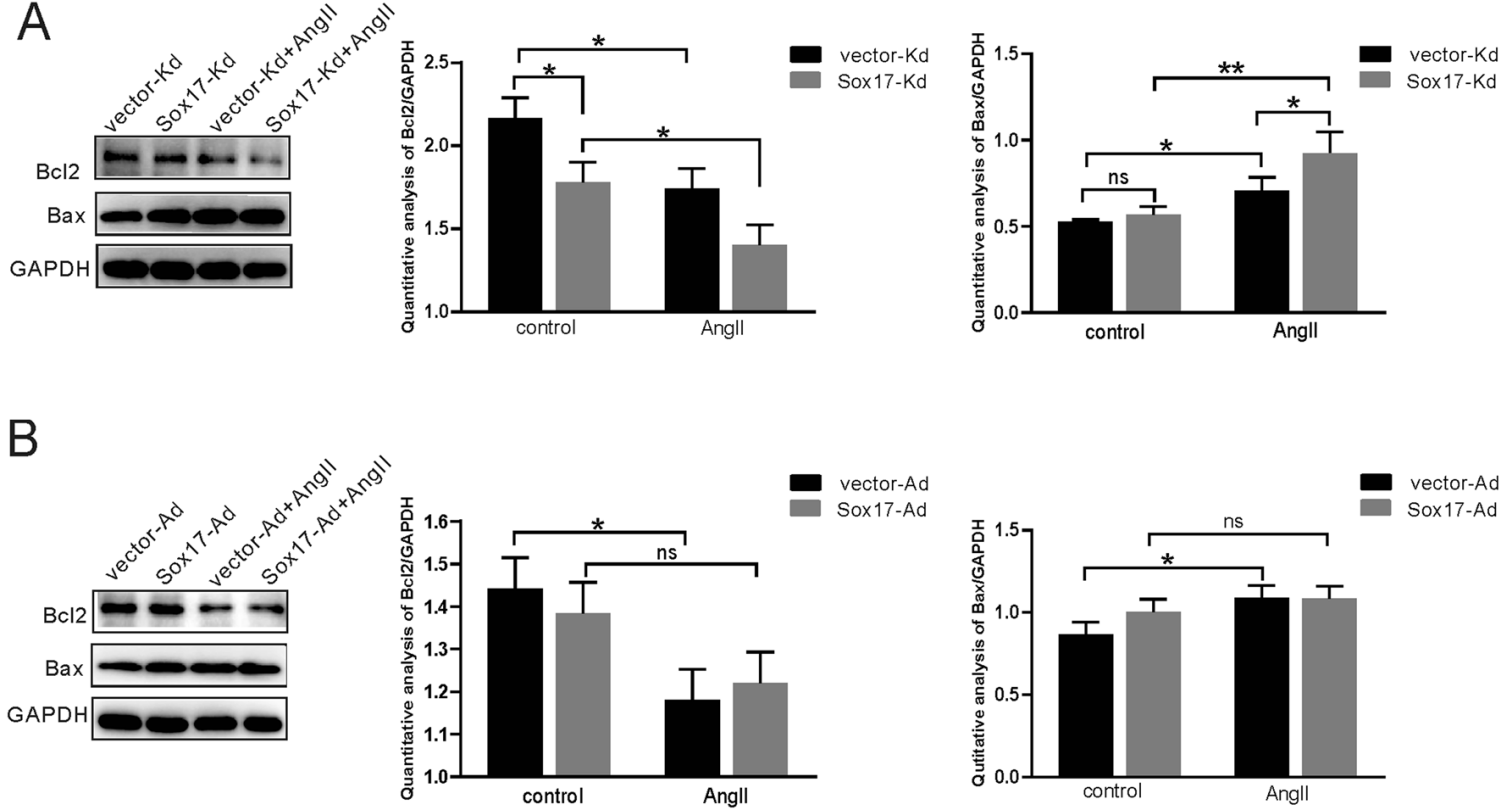
Effect of Sox17 on AngII-induced cell apoptosis. **A** Western blotting was used to detect the expression of Bax and Bcl-2 after the knockdown of Sox17 in the absence or presence of AngII. **B** Western blots were used to detect the expression of Bax and Bcl-2 after overexpression of Sox17 in the absence or presence of AngII. Data were expressed as mean ± SEM (*n* = 3). *p < 0.05, ** *p* < 0.01, two-way ANOVA with Tukey's multiple comparisons test. *ns* not significant, *AngII* angiotensin II, *control* without angiotensin II

We further investigated the changes of apoptosis-related proteins by western blotting. Knockdown of Sox17 reduced Bcl-2 expression compared to the control group. No significant difference was observed in the expression of Bax after silencing of Sox17 (Fig. 4A). There was no significant change in the expression of Bax and Bcl-2 after overexpression of Sox17 (Fig. 4B). Interestingly, after the AngII stimulation, the expression of Bax in the vector-Kd group was elevated, while the expression of Bcl-2 was decreased (Fig. 4A). The expression of Bax in the Sox17-Kd group was significantly increased compared with the vector-Kd group after AngII stimulation, and Bcl-2 was further decreased (Fig. 4A). However, under the AngII stimulation conditions, the expression of Bax and Bcl-2 in the Sox17-Ad group was steady compared to the vector-Ad group (Fig. 4B).

### Knockdown of Sox17 induced autophagy activation on HBMEC

**Fig. 5:**
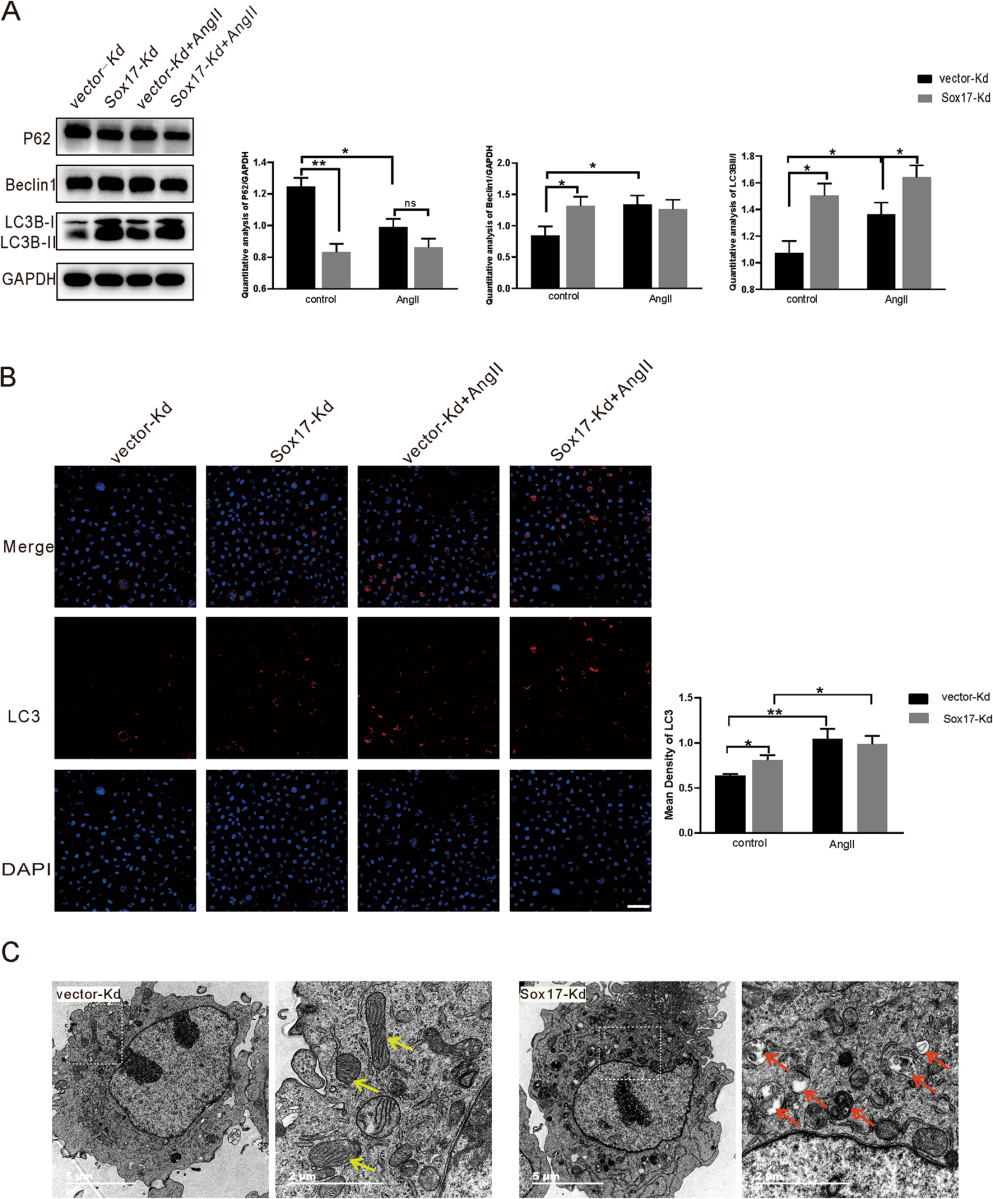
Knockdown of Sox17 induced autophagy of HBMEC. **A** Western blotting was used to detect the expression of P62, Beclin1, LC3 after the knockdown of Sox17 in the absence or presence of AngII. **B** Fluorescence microscopic images of LC3 in vector-Kd and Sox17-Kd groups in the absence or presence of AngII. Representative images of LC3 (red) and DAPI (blue) (*n* = 3; scale bar = 100 µm). **C** Representative images of transmission electron microscopy (TEM) showing autophagosomes in HBMECs after knockdown of Sox17, magnification × 23,000. In the ultrastructure of the HBMEC, yellow arrows point to the normal mitochondria, and red arrows indicate the representative autophagosomes. (*n* = 3; scale bar = 5 or 1 μm). *p < 0.05, ** *p* < 0.01, two-way ANOVA with Tukey's multiple comparisons test

Previous investigations demonstrated that autophagy plays a vital role in mediating cell apoptosis. In terms of the anti-apoptosis effects of Sox17 after exposure to Ang II challenge, we analyzed the expression of Sox17 and autophagy-related proteins by western blotting. We found that the knockdown of Sox17 led to increased expression of LC3-II/I, decreased P62, and increased Beclin1, suggesting enhanced autophagy. The expression of LC3-II/I in the Sox17-Kd group was significantly increased compared to the vector-Kd group during AngII stimulation (Fig. 5A). Consistent with these findings, immunofluorescence also validated that knockdown of Sox17 increased LC3 expression (Fig. 5B). Transmission fiber glass scans showed many visible autophagy bodies after silencing of Sox17, indicating enhanced autophagy in HBMEC (Fig. 5C).

### Ectopic expression of Sox17 suppressed HBMEC autophagy

**Fig. 6:**
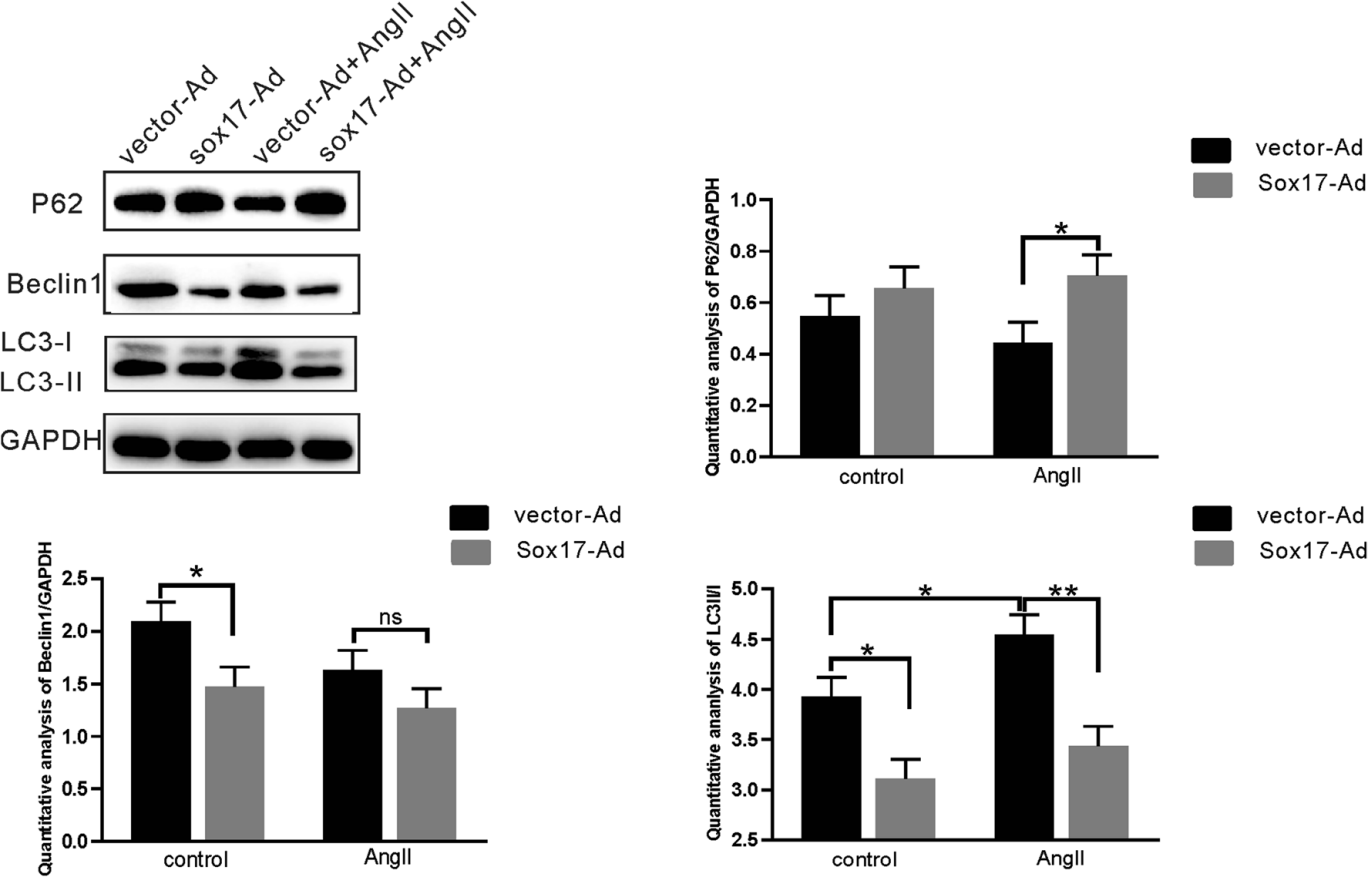
Overexpression of Sox17 reduced Autophagy. Representative western blots and quantification data of P62, Beclin1, LC3 after overexpression of Sox17. Data were expressed as mean ± SEM (*n* = 3). *p < 0.05, ** *p* < 0.01, two-way ANOVA with Tukey's multiple comparisons test. *ns* not significant, *AngII* angiotensin II, *control* without angiotensin II

To further determine the functional role of Sox17 on autophagy, we examined the expression of autophagy-related proteins (P62, Beclin1, and LC3-II/I) in the Sox17-Ad group, and overexpression of Sox17 inhibited the expression of LC3-II/I and Beclin1 (Fig. 6). Moreover, the expression of LC3-II/I in the Sox17-Ad group was significantly reduced compared with the vector-Ad group after AngII stimulation. The expression of P62 was significantly increased (Fig. 6).

### Transcriptome sequencing identified the connection between Sox17 and autophagy

**Fig. 7:**
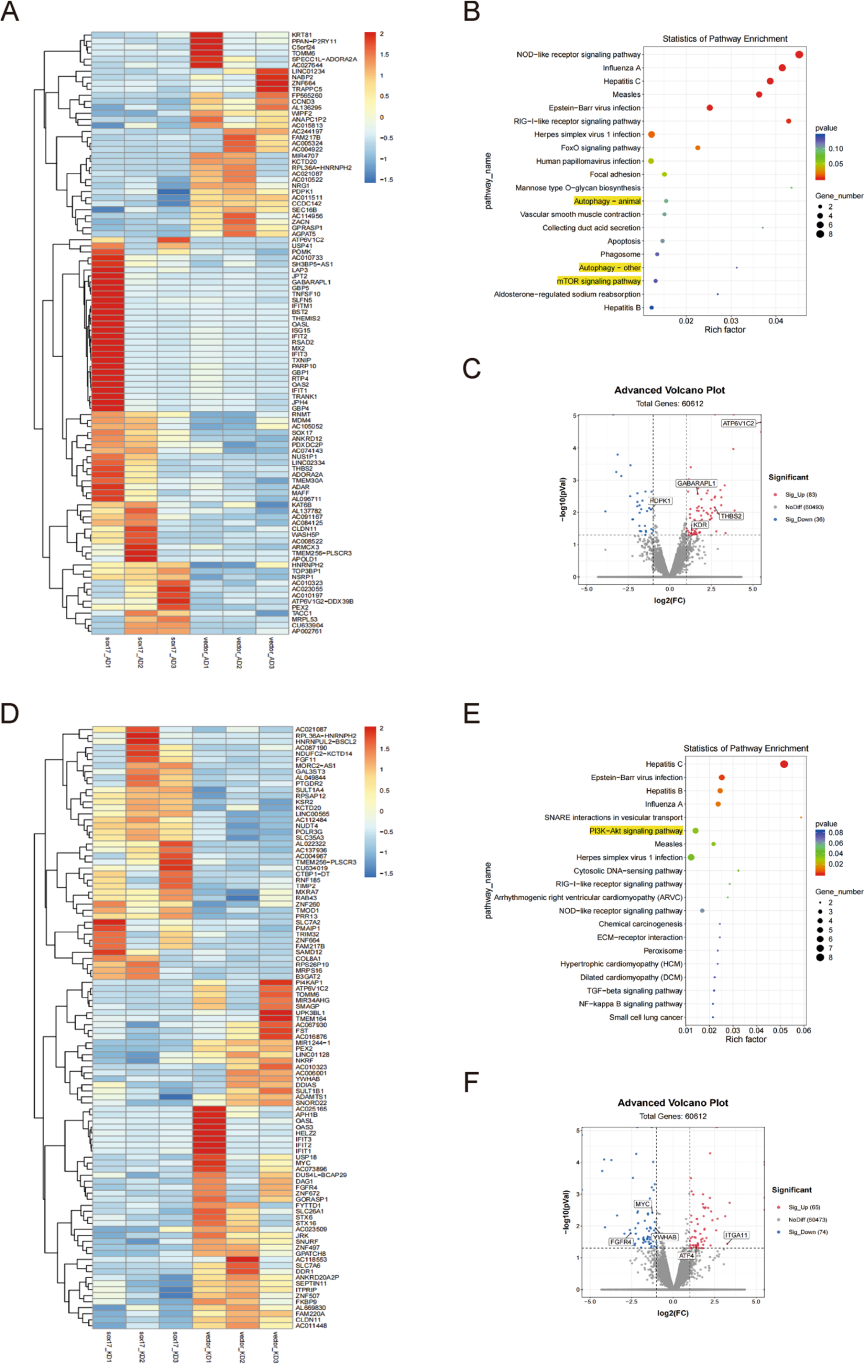
Genome-wide RNA-seq was used to examine the effect of Sox17 on gene expression. **A** Heatmap of differentially expressed genes in HBMECs between Sox17-Ad and vector-Ad groups. **B** KEGG enrichment of top 20 significant signaling pathways in the HBMECs with overexpression of Sox17. The size of the dots represents the gene number, and the color of the dots represents the p-value. **C** The volcano plot showed variance in gene expression concerning fold change and P-value. Each dot represents a separate transcript: red dots represent the up-regulated differentially expressed transcripts, blue dots represent the down-regulated differentially expressed transcripts, and grey dots represent not differentially expressed transcripts. **D** Heatmap of differentially expressed genes in HBMECs between Sox17-Kd and vector-Kd groups. **E** KEGG enrichment of the top 20 significant signaling pathways in Sox17 knockdown intervened HBMECs. **F** The volcano plot showed the genes with significant changes. The pathways accociated with autophagy were highlighted in yellow

To further illuminate the connection between Sox17 alteration and autophagy, we evaluated the RNA expression profiles after knockdown or overexpression of Sox17. Differentially expressed mRNA (DEmRNA) were obtained under the condition of fold change of > 2 or < 0.5 and *p* < 0.05. Heatmap analysis revealed differences in mRNA expression following overexpression of Sox17 (Fig. 7A). The volcano plot indicated that there were 83 up-regulated mRNAs and 36 down-regulated mRNAs, including ATP6V1C2, GABARAPL1, KDR, THBS2, and PDPK1 (Fig. 7C). KEGG analysis demonstrated that the DEmRNAs were primarily enriched in the NOD-like receptor signaling pathway (Fig. 7B). When Sox17 was knocked down, the heatmap and volcano plot illustrated 65 up-regulated genes and 74 down-regulated mRNA, especially ITGA11, ATF4, MYC, YWHAB, and FGFR4 (Fig. 7D, F). KEGG analysis showed that DEmRNA were mainly enriched in the PI3K-Akt signaling pathway and SNARE interactions in vesicular transport (Fig. 7E). Interestingly, regardless of whether Sox17 was overexpressed or knocked down, signaling pathways associated with autophagy consistently ranked among the top 20 significantly dysregulated pathways in the KEGG analysis. These pathways include the PI3K/AKT pathway, mTOR pathway, and autophagy pathway (Fig. 7).

## Discussion

The formation of IA is tightly modulated by various factors, ranging from environmental factors to genetics [29]. To build up the connection between external and intrinsic elements of IA, AngII was applied in hypertension modelas an inducer. We used AngII to challenge the HBMEC after knockdown or overexpression of Sox17 to observe the response in HBMEC. AngII is a vasoactive polypeptide that regulates various physiological functions such as vasoconstriction, fluid volume regulation, cardiac output, cell growth, and blood vessel wall integrity. The AngII type 1 receptor (AT1R) is considered to mediate the functions of AngII in the system. AT1R is found in various tissues, including vascular smooth muscle, endothelial cells, heart, brain, kidney, adrenal glands, and adipose tissue. Elevated AngII levels are closely related to the occurrence and rupture of abdominal aortic aneurysms and IA [30]. In previous research, AngII was used in vascular smooth muscle cell to explored its function, including inflammation and proliferation, which are involved in the pathology of hypertension and atherosclerosis [31]. Furthermore, the dysfunction of leukocytes, endothelium, white adipocytes and fibroblasts poses a risk for the development of aortic aneurysms under AngII induction. Reports have demonstrated that the infusion of AngII leads to the activation of iNOS/NF-κB in inflammatory leukocytes, resulting in an increase in the atherosclerotic lesion area of SHR rats [32]. Besides, white adipocytes and fibroblast have been reported to participate in the diameter regulation of the abdominal aorta following AngII infusion through direct interactions with AT1aR [33, 34]. AngII effect on human aortic endothelial cells (HAECs) induce oxidative stress, which increases ROS production, produces superoxides and hydrogen peroxide, affecting cell viability [8].

Actually, to explore the function of endothelial cells in intracranial aneurysm (IA) lesions, a 3-dimensional (3D) casted mold with endothelial cells was built to reveal the comprehensive gene expression profile [35]. Besides, 3D culture conditions have been shown to exhibit an expression profile more similar to that observed in native tissue, making it a valuable tool for investigating differential gene expression profiles compared to traditional 2D culture methods. 3D culture has been used in the mechanism research and drug development of heart disease and pulmonary hypertension [36, 37]. In the future, 3D spheroid of IA will be developed to further explore the pathogenesis of IA and identify potential targets for clinical drug interventions. In this study, we focus on the function of Sox17 in AngII induced hypertension model. Sox17 was observed to be specifically expressed in endothelial cells during vascular development of vascular system [38]. Further studies confirmed the key role of Sox17 in the cardiovascular system [15] and maintenance of the blood–brain barrier [39], as well as the differentiation of arteries [17, 40]. Hence, we chosen the HBMEC with AngII administration to build the cell model for observing the undying mechanisms after knockdown or overexpression of Sox17. Meanwhile, human genomic analysis has shown that the Sox17 site is one of the hot spots closely associated with intracranial aneurysms [13] and that deficiency of Sox17 leads to the formation of hereditary-related intracranial aneurysms induced by high blood pressure [4].

To further investigate the mechanism by which the Sox17 gene mediates hypertensive intracranial aneurysms, we established stable cell lines with knockdown or overexpression of Sox17 in HBMEC (Fig. 2). Subsequent studies have found that Sox17 knockdown exacerbated AngII-induced apoptosis of HBMEC (Figs. 3A, 4A). Apoptosis refers to the programmed cell death that is tightly controlled to maintain cellular homeostasis, and apoptosis is essential for cell survival. Previous studies have shown that AngII causes apoptosis of vascular endothelial cells [8, 41]. This study confirmed that AngII could up-regulate Bax expression and down-regulate Bcl-2 expression (Fig. 4A,B). Interestingly, the knockdown of Sox17 alone resulted in reduced Bcl-2 expression without Bax expression alteration (Fig. 4A) and no effect on apoptosis rate (Fig. 3A). However, the overexpression of Sox17 alone did not affect both the apoptosis-related proteins or the apoptosis rate (Figs. 3B, 4B). Bcl-2 plays a significant role in regulating apoptosis, and Bax is a pro-apoptotic protein [42]. Bcl-2 is one of the pleiotropic genes involved in apoptosis and autophagy [43]. Bcl-2/BclXL physically interacts with Beclin 1 through the BH3 domain, leading to Bcl-2-mediated suppression on autophagy [23, 44]. Sox17 knockdown leads to decreased Bcl-2 protein expression and increased Beclin1 expression, suggesting an enhancement of autophagy (Figs. 4A, 5A). This evidenced the Sox17 regulated the crosstalk between autophagy and apoptosis through Bcl-2 and Beclin1. Autophagy genes are involved in the execution of cell death. The crosstalk between apoptosis and autophagy is complicated [44, 45]. Apoptosis and autophagy are not mutually exclusive pathways. Their crosstalk has proved to be synergistic and confrontational. They shared many common molecular regulators. Under certain circumstances, autophagy is accompanied by cell death [46]. Our study found that the knockdown of Sox17 induced autophagy and apoptosis of human brain microvascular endothelial cells caused by AngII stimulation.

Autophagy plays an important role in paracrine regulation of endothelial vasoactive substances, which contributes to the development of vascular biology [28]. Disruption of autophagy is involved in the pathogenesis of the cardiovascular disease [47]. AngII can gradually induce autophagy, aging, and apoptosis of human umbilical vein endothelial cells [9]. In this study, we found that AngII induced autophagy in human brain microvascular endothelial cells (Figs. 5, 6). LC3 precursor is hydrolyzed by a cysteine protease called ATG4B and then converted to a proteolytic form termed LC3-I, which lacks C-terminal amino acids and is processed as a membrane-associated form termed LC3-II [48]. LC3-II/I protein ratio is an important indicator of autophagy [49]. Decreased expression of P62 is a canonical indicator of autophagic flux because it is involved in autophagic lysosomes degrading ubiquitinated proteins [50, 51]. The activity of the PI3K/AKT/mTOR pathway inhibits the autophagy process, which is closely associated with the prosurvival signals. The mammalian target of rapamycin (mTOR) is a crucial modulator of autophagy [52]. It has been shown that mTOR regulates the expression of Sox17 in endothelial cells [21]. That was supported by the transcriptome results, which revealed that the over expression of Sox17 in AngII induced HBMEC resulted in the regulation of KDR, PDPK1 and ATP6V1C2, genes that are associated with PI3K/AKT/mTOR pathway. Additonally, the knockdown of Sox17 regulated the genes ITGA11, ATF4, MYC, YWHAB, and FGFR4, which are involved in the PI3K/AKT/mTOR and autophagy pathways (Fig. 7). Besides, we found that knockdown of Sox17 in HBMEC up-regulated LC3-II and LC3-I expression, reduced the expression of P62, and increased the number of autophagosomes (Fig. 5). Overexpression of Sox17 can down-regulate LC3-II/I expression and increase P62 protein expression (Fig. 6). This suggested that Sox17 might modulate autophagy in vascular endothelial cells by mediating autophagic flux. Interestingly, we also found that silencing of Sox17 plus AngII stimulus increased the expression of LC3-II/I protein compared to the AngII-treated group. Moreover, we also confirmed that overexpression of Sox17 in HBMEC inhibited AngII-induced autophagy (Fig. 6). Finally, RNA-sequencing analysis also showed that Sox17 is closely related to autophagy gene including GABARAPL1 which is the essential for autophagosome maturation with LC3s (Fig. 7). Taken together, this study suggests that Sox17 might regulate the process of autophagy.

**Fig. 8:**
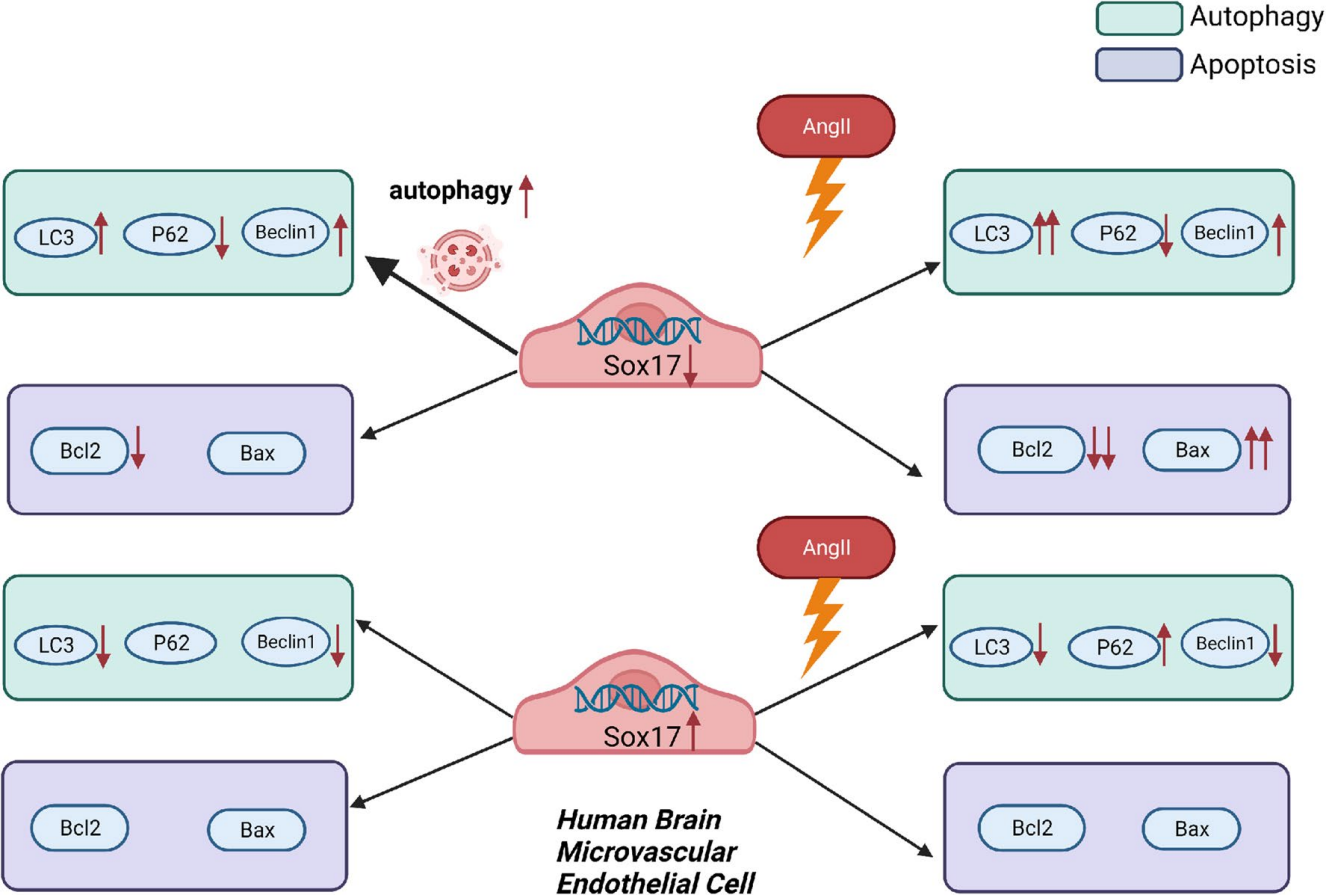
Schematic diagram illustrating the effects of Sox17 on HBMECs. The knockdown of Sox17 worsen the apoptosis and autophagy of HBMECs caused by angiotensin II, while overexpression of Sox17 had the opposite effect. The autophagosomes increased after the knockdown of Sox17 in HBMECs

In summary, our study described how the Sox17 gene alteration affects autophagy and apoptosis of HBMECs. Sox17 knockdown exacerbates autophagy and apoptosis of human brain microvascular endothelial cells induced by AngII stimulation, while overexpression of Sox17 can reverse this (Fig. 8). Our study revealed a previously unknown role of Sox17 in maintaining the function of vascular endothelial cells and demonstrated its impact on intracranial aneurysms. This findings suggest that Sox17 could serves as a promising therapeutic target for intracranial aneurysms. In clinical practice, it is necessary to control blood pressure in patients with familial IA, particualarly those with Sox17 gene defects. However, it is important to note that this study was conducted solely in vitro and focused on only a single cell line (HBMECs), which may be considered a potential limitation. Future research should encompass in vivo studies and investigations involving other cell lines to further validate and expand upon our findings.

## Data Availability

The data that support the findings of this study are available from the corresponding author upon reasonable request.

## Abbreviations

IA: Intracranial aneurysm
SAH: Subarachnoid haemorrhage
HBMEC: The human brain microvascular endothelial cell
AngII: Angiotensin II
GWAS: Genome-wide association studies
Sox: SRY Related-HMG box
VEGF: Vascular endothelial growth factor
RT: Room temperature
GFP: Green fluorescence protein
DAPI: 4′,6-Diamidino-2-phenylindole
BCA: Bicinchoninic acid
ECL: Enhanced chemiluminescence
PBS: Phosphate-buffered saline
PVDF: Polyvinylidene fluoride
RIPA: Radioimmunoprecipitation assay
BSA: Bovine serum albumin
TBST: Tris–HCl-Tween
CCK-8: Cell Counting Kit-8
PBS: Phosphatebuffer saline
PBST: Phosphate buffer saline-Tween
PE: Phycoerythrin
7-AAD: Annexin-V and 7-Amino-Actinomycin
3D: 3-Dimensional
P62: Nucleoporin p62
LC3: Microtubule-associated proteins 1 light chain 3
Bax: Bcl-2-associated X proteinand Bcl-2
Bcl-2: B-cell lymphoma 2
KEGG: Kyoto Encyclopedia of Genes and Genome
